# A Comparison of Parapoxviruses in North American Pinnipeds

**DOI:** 10.3389/fvets.2021.653094

**Published:** 2021-05-17

**Authors:** Helena Costa, Jörn Klein, Eva M. Breines, Hendrik H. Nollens, Keith Matassa, Mendy Garron, Padraig J. Duignan, Todd Schmitt, Tracey Goldstein, Morten Tryland

**Affiliations:** ^1^Department of Arctic and Marine Biology, UiT The Arctic University of Norway, Tromsø, Norway; ^2^Faculty of Veterinary Medicine of University of Lisbon, Lisbon, Portugal; ^3^Faculty of Health and Social Sciences, University of South-Eastern Norway, Notodden, Norway; ^4^Pacific Marine Mammal Center, Laguna Beach, CA, United States; ^5^Ocean Animal Response and Research Alliance, Dana Point, CA, United States; ^6^National Oceanic and Atmospheric Administration (NOAA), Greater Atlantic Regional Fisheries Office, Gloucester, MA, United States; ^7^The Marine Mammal Center, Sausalito, CA, United States; ^8^SeaWorld Parks and Entertainment, San Diego, CA, United States; ^9^One Health Institute and Karen C. Drayer Wildlife Health Center, University of California, Davis, Davis, CA, United States

**Keywords:** parapoxvirus, seal parapoxvirus, pinniped, skin lesion, sealpox

## Abstract

Parapoxviruses cause nodular lesions on the skin and mucosal membranes of pinnipeds and infections by these viruses have been documented worldwide. Seal parapoxvirus is currently classified as a tentative species of the *Parapoxvirus* genus. Tissue or swab samples were analyzed from 11 pinnipeds of different host species undergoing rehabilitation on the east and west coasts of the United States of America (USA) that were positive for parapoxvirus. The aim of the study was to compare parapoxvirus sequences of fragments of the *B2L, DNA polymerase, GIF* and *viral interleukin-10 ortholog* (*vIL-10)* genes and to examine the evolutionary relationship between viruses detected in different pinniped species and at different locations with other members of the *Parapoxvirus* genus, such as Orf virus (ORFV), Bovine papular stomatitis virus (BPSV) and Pseudocowpox virus (PCPV). The sequence analysis showed that the parapoxvirus sequences from the pinnipeds differed significantly from those found in terrestrial hosts and that they formed a separate cluster within the genus. Our results suggest that transmission of the same parapoxvirus strain is possible between different species, including between members of different families (phocids and otariids). Animals belonging to the same species but living in distant geographic locations presented genetically distant parapoxviruses. The findings of this study demonstrate that sealpox lesions in pinnipeds of different species are caused by viruses that belong to the *Parapoxvirus* genus but have significant genetic differences compared to the established virus species in terrestrial hosts, thus strongly supporting the classification of pinniped parapoxvirus as a new species of the genus.

## Introduction

Parapoxviruses cause nodular lesions on the skin and mucosal membranes of pinnipeds and pox infections have been documented worldwide in animals admitted to rehabilitation facilities (1–8) and in free-ranging animals (8–12). Pinniped parapoxviruses are currently classified as the tentative species “seal parapoxvirus” in the *Parapoxvirus* genus, of which the recognized members include Orf virus (ORFV), Bovine papular stomatitis virus (BPSV), Pseudocowpox virus (PCPV) and parapoxvirus of red deer in New Zealand (PVNZ) (13). Other species such as the camel contagious ecthyma virus, chamois contagious ecthyma virus (14) and a parapoxvirus of horses (15) have also been proposed to be included in this genus.

Pinnipeds infected with parapoxvirus typically develop one or more firm skin nodules, 1 to 3 cm in diameter, usually affecting the head, neck, flippers and thorax (16). These lesions frequently heal spontaneously, resolving, in most cases, in 4–6 weeks (17). Besides being transmissible between individuals (1, 3, 18), parapoxviruses are also zoonotic, and typical parapoxvirus lesions have been described in people who had contact with parapoxvirus infected pinnipeds (2, 19). Parapoxvirus infections are diagnosed through clinical evaluation of skin and mucosal lesions, virus isolation, electron microscopy (20), serological tests (21) and molecular detection (16, 22).

The parapoxvirus core genes are responsible for genome replication, transcription and virion assembly and are highly conserved among the different species (23). The genes located near the genomic termini encode for viral functions which play a role in pathogenesis, tissue tropism, virulence and viral-host interactions (24). The conserved core genes, such as the parapoxvirus major envelope gene (*B2L* gene) and *DNA polymerase* gene, are therefore more useful for comparing sequences within the *Parapoxvirus* genus (22) while more variable terminal genomic regions such as the virulence genes viral interleukin-10 ortholog (*vIL-10)* and *GIF* (25, 26) are useful for the separation of parapoxvirus species and strains.

This study aimed to compare the evolutionary relationship of pinniped parapoxvirus to other members of the genus that infect terrestrial mammals and evaluate if different strains of parapoxvirus are circulating in pinniped populations along the Atlantic and Pacific north American coasts.

## Materials and Methods

### Clinical Cases

**Figure 1:**
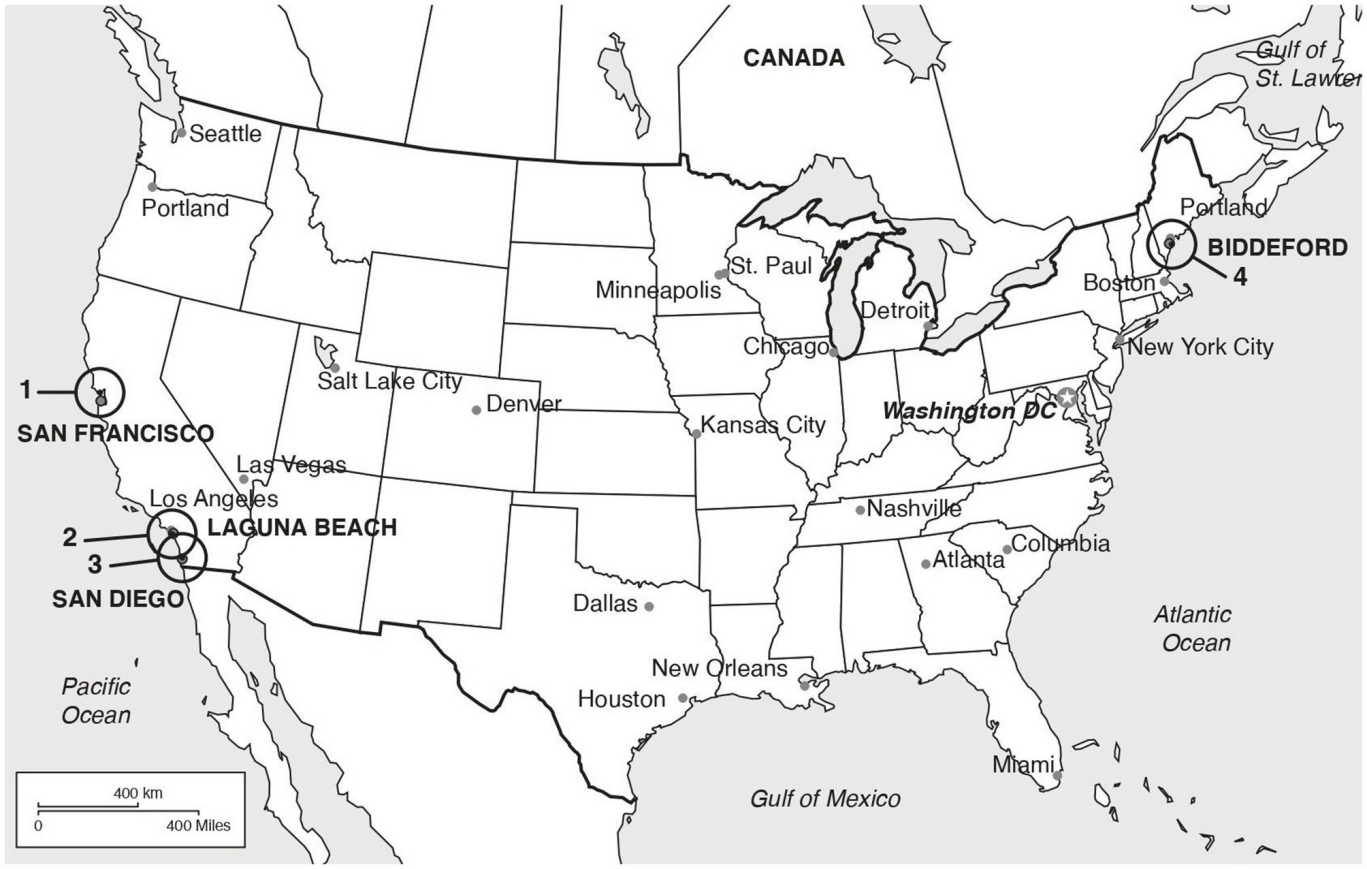
Locations of the rehabilitation facilities where the animals were sampled. (1) The Marine Mammal Center (TMMC; Sausalito, San Francisco); (2) Pacific Marine Mammal Cente (PMMC; Los Angeles); (3) Sea World (San Diego); (4) Marine Animal Rehabilitation Center, University of New University of New England (Biddeford).

Skin and ocular swab samples were analyzed from eleven pinnipeds of five different species, listed below, that were confirmed with parapoxvirus infection by polymerase chain reaction (PCR) between 2009 and 2018. The animals were in care at four different rehabilitation facilities in the United States of America (USA). Samples from the east coast were obtained through the Marine Animal Rehabilitation Center, University of New England, Biddeford, Maine, New England, and included two gray seals (*Halichoerus grypus*), UiT1 and UiT2, and two Atlantic harbor seals (*Phoca vitulina vitulina*), UiT4 and UiT5. Pinnipeds endemic to the west coast were sampled at The Marine Mammal Center (TMMC; Sausalito, San Francisco), Pacific Marine Mammal Center (PMMC; Laguna Beach, Orange County) and Sea World (San Diego), all in the state of California (Figure 1). The samples included three Pacific harbor seals (*Phoca vitulina richardii*), UiT6, UiT7 and UiT8, three California sea lions (*Zalophus californianus*), UiT9, UiT11 and UiT12, and one northern elephant seal (*Mirounga angustirostris*), UiT10. All animal handling and sample collection procedures were done and under the supervision of the veterinary team and respecting animal welfare guidelines.

### Parapoxvirus Detection

Deoxyribonucleic acid (DNA) was extracted from the clinical samples (Maxwell® 16 Buccal Swab LEV DNA Purification Kit; Promega, Madison, WI, USA), and DNA quality assessed using the Nanodrop 2000 spectrophotometer (Thermo Fisher Scientific™, Portsmouth, NH, USA). Four parapoxvirus specific PCRs were performed, using a Perkin Elmer GeneAmp® PCR System 9700 (Perkin Elmer Corp., Shelton, CT, USA), targeting four different gene regions: the *B2L* gene, with primers PPP-1 and PPP-4, based on the *B2L* gene sequence of ORFV (strain NZ2) and using the protocol described by Inoshima et al. (22); the *GIF* gene, encoding a protein inhibiting the granulocyte-macrophage-colony-stimulating factor and interleukin-2, with the primers GIF5/GIF6 and the *vIL-10* gene, with primers vIL-10-3/vIL-10-4, using the protocol described by Klein and Tryland (27); and the *DNA polymerase* gene, with the primers PPV/DNApol-F and PPV/DNApol-R, using the protocol described by Bracht et al. (28) but with an increased annealing temperature (65°C). PCR was performed in a final volume of 25 μL using 1 μL of the forward (25 μM) and reverse (25 μM) primers, 10 μL of JumpStart™ REDTaq® ReadyMix™ (Sigma-Aldrich Co. LLC), 8 μL of DNAse-free water and 5 μL of extracted DNA from seal samples. Water was used as non-template control and ORFV DNA was obtained from a goat kid (*Capra hircus*) diagnosed with contagious ecthyma and verified ORFV infection (Norwegian Veterinary Institute, Tromsø, Norway) was used as a positive control. Amplified DNA fragments were separated by horizontal electrophoresis of 10 μL of the PCR product in 1% agarose, containing 5% of gelRED™, visualized under ultraviolet light and photographed using a gel documentation system (Bio-Rad Laboratories). PCR products were cleaned by ExoSAP-IT® (Applied Biosystems™) and sequenced in both directions (BigDye® Terminator Version 3.1 cycle sequencing kit, Applied Biosystems™) in an Applied Biosystems 3130 XL Genetic Analyzer (Applied Biosystems™).

### Sequence Analysis

Raw sequences were edited using Chromas software (Version 2.6.6; Technelysium Pty Ltd., Tewantin, Qld., Australia). Nucleotide sequences were subjected to a Basic Local Alignment Search Tool (BLAST) search (http://blast.ncbi.nlm.nih.gov/Blast.cgi) for comparison with other parapoxvirus sequences available in the GenBank NCBI database (29).

Sequences were aligned along with comparable parapoxvirus sequences from pinniped and terrestrial hosts (sheep, goats, muskoxen, cattle, and humans), from GenBank database using MEGAX (30) and the ClustalW algorithm. The phylogenetic relationships were estimated using two trees based on the *B2L* and *DNA polymerase* gene regions. Trees were constructed using the maximum-likehood method (31) based on the calculation of the genetic distances between pairs of sequences using the Tamura 3-parameter model (32). The statistical support for both trees was provided by 1,000 bootstrap replicates with the respective percentages indicated on the branches.

## Results

**Figure 2:**
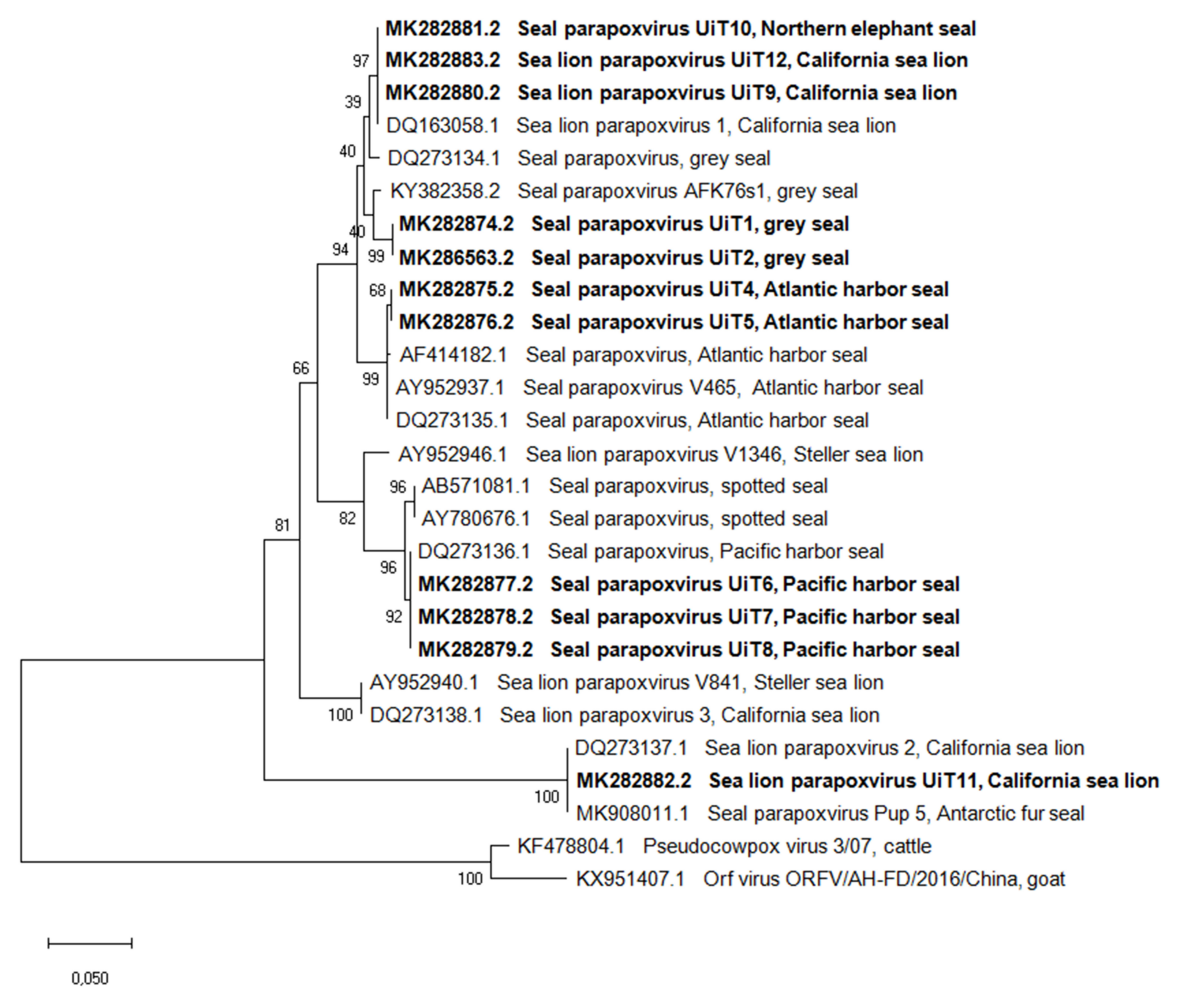
Phylogenetic tree based on the partial nucleotide sequences of the *B2L* gene obtained in this study compared with corresponding DNA sequences from parapoxviruses published in GenBank. Isolates are described by GenBank accession number, parapoxvirus species and host species. The phylogenetic tree was constructed using the maximum-likehood method based on the calculation of the genetic distances between pairs of sequences using the Tamura 3-parameter model. The statistical support for the tree was provided by 1,000 bootstrap replicates with the respective percentages indicated on the branches. The scale bar corresponds to 0.05 aa substitutions per site. Sequences obtained in this study are shown in bold.

Amplicons of the expected size (594 bp) were generated from all the cases when targeting the parapoxvirus *B2L* gene. The phylogenetic tree based on the *B2L* gene (Figure 2) showed, with two exceptions, specific clustering of viruses that paralleled the phylogenetic relatedness of their host species. The tree showed that the two Atlantic harbor seals formed a separate cluster with other Atlantic harbor seals, separating them from the cluster formed by the Pacific harbor seals (from this study and from GenBank database). All the obtained samples, with exception of the sequence of the California sea lion UiT11, shared a nucleotide identity of 91–100% with the other seal and sea lion parapoxvirus sequences in the GenBank database (Supplementary Table 1). The sequence from the California sea lion UiT11, however, clustered with another California sea lion parapoxvirus (accession number DQ273137.1) and a parapoxvirus sequence from an Antarctic fur seal pup (accession number MK908011.1) (33) and these sequences shared only 84–86% nucleotide identity with the rest of the generated sequences in this study. The other exception to the clustering according to pinniped species was the sequence from the Northern elephant seal (UiT10) that clustered with three sequences from California sea lion parapoxvirus (UiT12, UiT9, and DQ163058.1) in the B2L tree.

**Figure 3:**
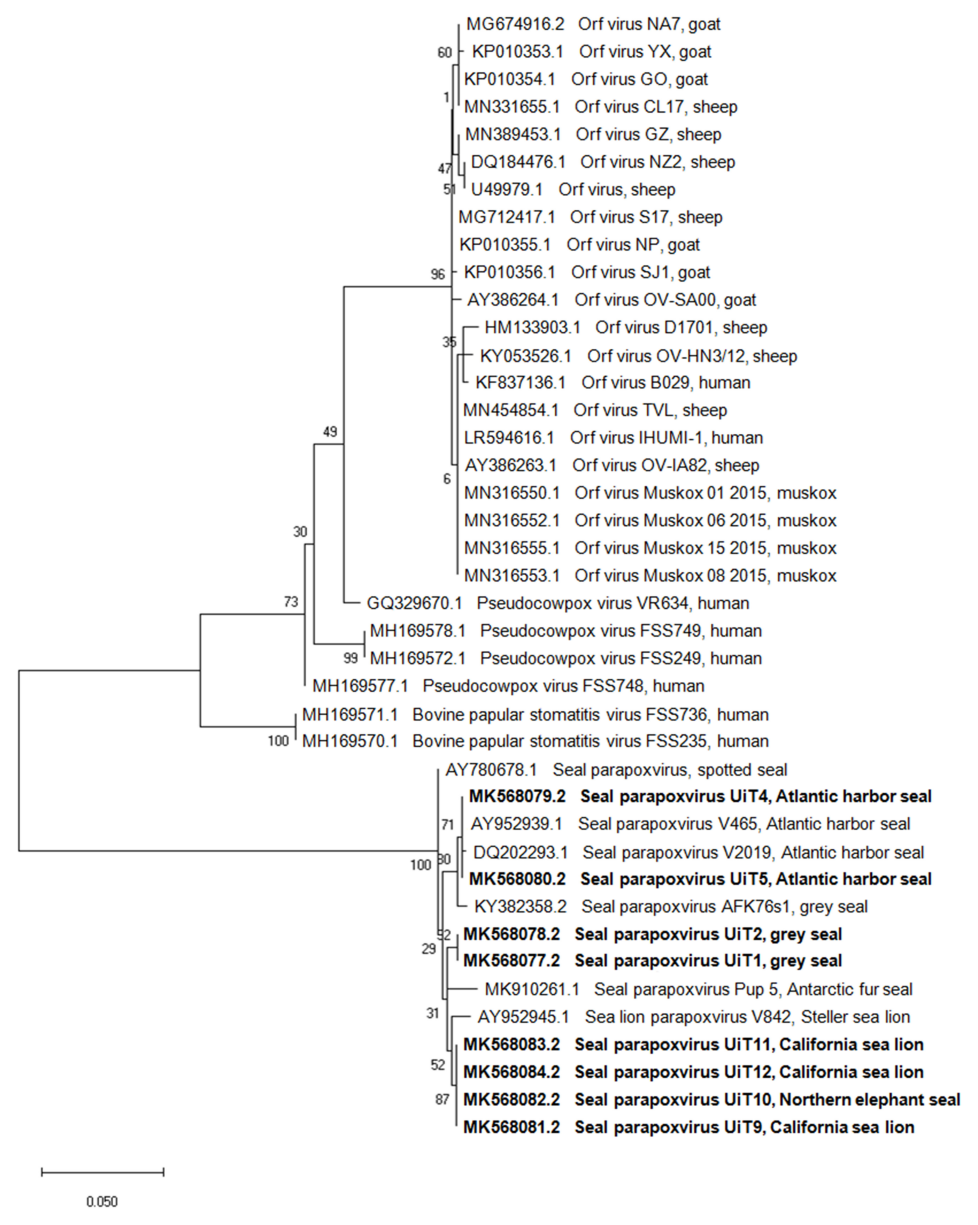
Phylogenetic tree based on the partial nucleotide sequences of the *DNA polymerase* gene obtained in this study compared with corresponding DNA sequences from parapoxviruses published in GenBank. Isolates are described by GenBank accession number, parapoxvirus species and host species. The phylogenetic tree was constructed using the maximum-likehood method based on the calculation of the genetic distances between pairs of sequences using the Tamura 3-parameter model. The statistical support for the tree was provided by 1,000 bootstrap replicates with the respective percentages indicated on the branches. The scale bar corresponds to 0.05 aa substitutions per site. Sequences obtained in this study are shown in bold.

Amplicons of the *DNA polymerase* gene (536 bp) were generated from all the cases except from the three Pacific harbor seals. Blast analysis of the DNA polymerase sequences from the eight individuals showed a nucleotide identity of 98–100% with the other seal and sea lion parapoxvirus sequences, except for a sequence from a Steller sea lion, *Eumetopias jubatus* (GenBank accession number AY952942.1) which had only 78%-80% similarity (Supplementary Table 2). The phylogenetic tree based on the *DNA polymerase* gene sequences (Figure 3) also presented separated clusters according to pinniped species, with the exception of the Northern elephant seal (UiT10) that clustered together with the California sea lions (UiT9, UiT11, UiT12).

The topology of both trees of the *B2L* and *DNA polymerase* gene sequences showed that while all sequences were most closely related to the other viruses in the *Parapoxvirus* genus, there was a clear separation into two distinct clusters based on the parapoxviruses associated with terrestrial host (ORFV, PCPV and BPSV) and pinniped host parapoxviruses. The gene sequences obtained in this study had 79–84% and 80–82% similarity to parapoxvirus from terrestrial hosts, for the *B2L* and the *DNA polymerase* genes, respectively.

The PCRs targeting the *GIF* and *vIL-10* genes failed to produce any amplicons of the expected size (408 and 300 bp).

## Discussion

Our findings demonstrate that sealpox lesions in pinnipeds of different species are caused by viruses that belong to the genus *Parapoxvirus* but are different from the viruses that infect terrestrial hosts, ORFV, BPSV and PCPV. Our results strongly support the classification of pinniped parapoxvirus as a new species within the genus. Considering that this virus has already been detected in members of all pinniped families, i.e., *Phocidae* (true seals), *Otariidae* (eared seals) and *Odobenidae* (walrus) (34), we suggest the use of the nomenclature “pinniped parapoxvirus” for this species rather than the previously used nomenclatures of “seal parapoxvirus” or “sealpox virus.”

In our study, animals belonging to the same species but living in distant geographic locations presented genetically distant parapoxviruses. The isolates from the Atlantic harbor seals (UiT4, UiT5) and Pacific harbor seals (UiT6, UiT7, UiT8) clustered separately. Also, the isolates from the two gray seals (UiT1 and UiT2) clustered separately from a gray seal isolate from Germany (KY382358.2). This indicates the existence of different virus variants in different subspecies of pinnipeds, suggesting co-evolution of the virus with the host, likely reflecting the great geographical distances that separate these populations and the unlikelihood of contact between them. Additional studies on the phylogenetic relationships between isolates of different populations of different pinniped species would be of interest to better understand the circulation and transmission of this virus in the wild.

Our phylogenetic comparisons were consistent with findings by Nollens et al. (21) who reported the existence of different parapoxvirus strains circulating in the California sea lion population (Sea Lion Parapoxvirus-1, 2, 3; SLPV-1, 2, 3). We detected two different strains in our sampled animals: two California sea lions (UiT9 and UiT10) with SLPV-1 and one California sea lion (UiT11) with SLPV-2. The SLPV-2 strain infecting UiT11 was the most divergent from all other pinniped parapoxviruses, which is also consistent with Nollens et al. (21) results.

Interestingly, the sequence from the Northern elephant seal (UiT10) clustered with sequences from California sea lions that were housed in the same facility and samples were collected during the same month. This suggests that it is likely that the virus was transmitted between the two species in the rehabilitation facility. A previous report of a parapoxvirus outbreak in another facility also suggested transmission of a parapoxvirus from harbor seals to California sea lions and northern elephant seals based on clinical lesions, but no viral sequences were obtained to confirm the suspicion (1). Our findings suggest that transmission of the same parapoxvirus strain is possible between different species, including between members of different families i.e., phocids (northern elephant seals) and otariids (California sea lions). This possibility is of note to marine mammal rehabilitation managers as care should be taken to prevent introduction of novel pathogens to wildlife that are intended for reintroduction.

Of the four gene regions that were targeted, the primers targeting the *B2L* gene (PPP-1 and PPP-4) was the only one that amplified a DNA fragment from all cases, likely due to the highly conserved nature of this gene region (22). The primers targeting the *GIF* and *vIL-10* gene regions were designed based on sequences from ORFV isolates (27) but they did not amplify fragments from any of our pinniped samples. The *GIF* gene is located in the right terminal genome region (35) and many members of the subfamily *Chordpoxvirinae* show genetic rearrangement at the terminal genome sequences. This is thought to be an evolutionary phenomenon, allowing them to adapt to changes in the host's immune response (36). Rziha et al. (37) also reported rearrangement of duplications in the inverted terminal repeat of the *vIL-10* gene. Since *vIL-10* is a major virulence factor of parapoxviruses, it needs to have a close sequence similarity to the host's IL-10 (27). Therefore, it is likely that parapoxviruses that infect pinnipeds have a different *vIL-10* gene region than a parapoxvirus that infects terrestrial hosts. The sequence differences likely explain the negative PCR results and provide additional support for pinniped parapoxviruses being classified as separate members of the genus. The results also demonstrate that PCRs targeting the *GIF* and the *vIL-10* genes will be of less value for the detection of pinniped parapoxviruses.

It is unclear why *DNA polymerase* gene fragments were not amplified in the samples from the three Pacific harbor seals (UiT6, UiT7, and UiT8). These primers were designed based on genomic sequences of ORFV and BPSV but were successfully used by Bracht et al. (28) to detect parapoxvirus sequences in samples from Steller sea lions, spotted seals (*Phoca largha*) and one Atlantic harbor seal and from California sea lions (UiT9, UiT11, UiT12), a northern elephant seal (UiT10) and gray seals (UiT1, UiT2) in our study. The failure of detecting the parapoxviruses in samples from Pacific harbor seals may indicate species-specific sequence differences in that gene region, but the result should be interpreted carefully since extraction contaminants could have inhibited the PCR reaction.

It is common to house pinnipeds of different species in the same rehabilitation facility. Even if the animals are generally maintained in separate pens, parapoxviruses are highly resistant in the environment and may be transported accidentally between animals by care givers or on equipment. Many of the patients in rehabilitation facilities have significant diseases or malnutrition as the primary reason for admission which, together with the stress of the capture, transport and captivity, may cause immune suppression. The presence of active sealpox cases and difficulty of hygienization of certain surfaces (i.e., porous concrete walls) can lead these immuno-compromised animals to be more prone to infection. Further, it is important to keep in mind that parapoxviruses are zoonotic agents (38) that upon contact may be transferred to animal keepers and visitors of facilities that care for pinnipeds. Therefore, it is vital that quarantine procedures, virucidal cleaning procedures (e.g., bleach) and/or separation by animal species should be in place in facilities to reduce exposure to and introduction of novel virus strains into immunologically naïve animals, prevent disease outbreaks and zoonotic transmission.

## Data Availability Statement

The datasets presented in this study can be found in online repositories. The names of the repository/repositories and accession number(s) can be found at: https://www.ncbi.nlm.nih.gov/genbank/, MK282874.2, MK286563.2, MK282875.2, MK282876.2, MK282877.2, MK282878.2, MK282879.2, MK282880.2, MK282881.2, MK282882.2, MK282883.2, MK568077.2, MK568078.2, MK568079.2, MK568080.2, MK568081.2, MK568082.2, MK568083.2, MK568084.2.

## Ethics Statement

Ethical review and approval was not required for the animal study because the samples used in this study were sampled from patients undergoing rehabilitation in wildlife hospitals. The sample collection procedures were done under the supervision of the veterinary team and aiming for the diagnostics and subsequent treatment of the patients.

## Author Contributions

MT and TG: conceptualization. JK: formal analysis. HC and EB: investigation. HC: data curation, writing—original draft preparation, and visualization. HC, MT, TG, JK, HN, KM, MG, PD, and TS: writing—review and editing. MT: supervision, project administration, funding acquisition, methodology, and resources. All authors have read and agreed to the published version of the manuscript.

## Conflict of Interest

The authors declare that the research was conducted in the absence of any commercial or financial relationships that could be construed as a potential conflict of interest.
