# Supplementary material for: A Comparison of Parapoxviruses in North American Pinnipeds
Source: Front Vet Sci. 2021 May 17;8:653094. doi: 10.3389/fvets.2021.653094 (PMC8165162; doi:10.3389/fvets.2021.653094)
Supplement: Supplementary file 1 [file Table_1.DOCX]

|  | (*) UiT1  **Grey seal** | (*) UiT2  **Grey seal** | (*) UiT4  **Atlantic harbor seal** | (*) UiT5  **Atlantic harbor seal** | (*) UiT6  **Pacific harbor seal** | (*) UiT7  **Pacific harbor seal** | (*) UiT8  **Pacific harbor seal** | (*) UiT9  **California sea lion**  (SLPV-1) | (*) UiT10  **Northern elephant seal** | (*) UiT11  **California sea lion**  **(SLPV-2)** | (*) UiT12  **California sea lion**  (SLPV-1) |
| --- | --- | --- | --- | --- | --- | --- | --- | --- | --- | --- | --- |
| DQ273134.1  **Grey seal** | 98.65% | 98.65% | 96.48% | 96.35% | 93.93% | 93.92% | 93.96% | 99.02% | 99.03% | **85.01%** | 99.03% |
| KY382358.2  **Grey seal** | 98.42% | 98.42% | 97.08% | 97.13% | 93.36% | 93.35% | 93.39% | 98.04% | 98.05% | **85.63%** | 98.05% |
| AY952937.1  **Atlantic harbor seal** | 96.39% | 96.39% | 99.81% | 99.81% | 93.36% | 93.35% | 93.39% | 96.87% | 96.88% | **84.39%** | 96.88% |
| DQ273135.1  **Atlantic harbor seal** | 96.39% | 96.39% | 99.81% | 99.81% | 93.36% | 93.35% | 93.39% | 96.87% | 96.88% | **84.39%** | 96.88% |
| AF414182.1  **Atlantic harbor seal** | 96.16% | 96.16% | 99.61% | 99.62% | 93.16% | 93.15% | 93.19% | 96.67% | 96.69% | **84.19%** | 96.68% |
| DQ273136.1  **Pacific harbor seal** | 93.45% | 93.45% | 93.18% | 93.46% | 100.00% | 100.00% | 100.00% | 93.74% | 93.76% | **84.38%** | 93.75% |
| AB571081.1  **Spotted seal** | 93.00% | 93.00% | 92.40% | 92.69% | 98.83% | 98.83% | 98.83% | 93.35% | 93.37% | **84.38%** | 93.36% |
| AY780676.1  **Spotted seal** | 93.00% | 93.00% | 92.40% | 92.51% | 98.83% | 98.83% | 98.83% | 93.35% | 93.37% | **84.38%** | 93.36% |
| AY952946.1  **Steller sea lion** | 92.78% | 92.78% | 92.40% | 92.69% | 95.31% | 95.30% | 95.33% | 93.74% | 93.76% | **84.79%** | 93.75% |
| AY952940.1  **Steller sea lion** | 92.78% | 92.78% | 92.79% | 92.88% | 91.02% | 91.00% | 91.05% | 93.74% | 93.76% | **85.60%** | 93.75% |
| DQ163058.1  **California sea lion**  (SLPV-1) | 97.52% | 97.52% | 96.68% | 96.55% | 93.74% | 93.73% | 93.76% | 100.00% | 100.00% | **84.80%** | 100.00% |
| DQ273138.1  **California sea lion**  (SLPV-3) | 92.78% | 92.78% | 92.79% | 92.87% | 91.02% | 91.00% | 91.05% | 93.74% | 93.76% | **85.60%** | 93.75% |
| DQ273137.1  **California sea lion**  **(SLPV-2)** | **86.00%** | **86.00%** | **84.44%** | **84.66%** | **83.92%** | **83.92%** | **83.85%** | **84.71%** | **84.02%** | **100.00%** | **84.71%** |
| MK908011.1  **Antarctic fur seal**  **(SLPV-2)** | **85.78%** | **85.78%** | **84.11%** | **84.34%** | **84.11%** | **84.11%** | **84.11%** | **84.49%** | **84.49%** | **99.38%** | **84.49%** |
| KF478804.1  **PCPV - Cattle** | 83.85% | 79.61% | 78.99% | 82.00% | 78.17% | 81.63% | 78.25% | 78.32% | 78.40% | 78.03% | 78.36% |
| KT935589.1  **ORFV - Goat** | 80.85% | 78.82% | 78.79% | 78.96% | 78.28% | 78.28% | 78.25% | 78.04% | 78.02% | 77.66% | 77.97% |

**Supplementary Table 1.** The BLAST nucleotide identity percentage between the sequences of the *B2L* gene obtained in this study (*) and corresponding parapoxvirus sequences from pinniped and terrestrial hosts published in GenBank database. Highlighted are the nucleotide identity percentages between the California sea lion UiT11 and the available sequences in Genbank database and between the California sea lion D1273137.1, the Antarctic fur seal MK908011.1 and the sequences obtained in this study.
