# Supplementary material for: A Comparison of Parapoxviruses in North American Pinnipeds
Source: Front Vet Sci. 2021 May 17;8:653094. doi: 10.3389/fvets.2021.653094 (PMC8165162; doi:10.3389/fvets.2021.653094)
Supplement: Supplementary file 2 [file Table_2.DOCX]

|  | (*) UiT1  **Grey seal** | (*) UiT2  **Grey seal** | (*) UiT4  **Atlantic harbor seal** | (*) UiT5  **Atlantic harbor seal** | (*) UiT9  **California sea lion** | (*) UiT10  **Northern elephant seal** | (*) UiT11  **California sea lion** | (*) UiT12  **California sea lion** |
| --- | --- | --- | --- | --- | --- | --- | --- | --- |
| KY382358.2  **Grey seal** | 98.57% | 98.57% | 99.40% | 99.41% | 98.38% | 98.34% | 98.34% | 98.36% |
| AY952939.1  **Atlantic harbor seal** | 98.57% | 98.57% | 100.00% | 100.00% | 98.58% | 98.54% | 98.55% | 98.57% |
| DQ202293.1  **Atlantic harbor seal** | 98.37% | 98.37% | 99.80% | 99.80% | 98.38% | 98.34% | 98.34% | 98.36% |
| AY780678.1  **Spotted seal** | 99.18% | 99.18% | 99.00% | 99.02% | 99.19% | 99.17% | 99.17% | 99.18% |
| AY952945.1  **Steller sea lion** | 98.78% | 98.78% | 98.20% | 98.23% | 98.99% | 98.96% | 98.96% | 98.98% |
| AY952942.1  **Steller sea lion** | **77.96%** | **77.96%** | **80.43%** | **78.54%** | **78.09%** | **79.69%** | **80.04%** | **80.04%** |
| GQ329670.1; MH169578.1; MH169572.1; MH169577.1  **PCPV -Humans** | 81.84% | 81.84% | 81.50% - 82.04% | 81.50%- 82.48% | 81.02%-81.74% | 81.38%-82.01% | 81.37%-82.16% | 81.53%-82.27% |
| MG674916.2; KP010353.1; KP010354.1; KP010355.1; KP010356.1; AY386364.1  **ORFV - Goats** | 81.84% | 81.84% | 81.84%-82.24% | 82.21%-82.48% | 81.54%-81.74% | 81.80%-82.01% | 81.78%-82.19% | 81.89%-82.30% |
| MN331655.1; MN389453.1; DQ184476.1; U49979.1; MG712417.1; HM133903.1; KY053526.1; MN454854.1; AY386263.1  **ORFV- Sheep** | 81.02%-81.84% | 81.02%-81.84% | 81.44%-82.24% | 81.69%-82.48% | 80.93%-81.74% | 81.17%-82.01% | 81.37%-82.19% | 81.48%-82.30% |
| KF837136.1; LR594616.1  **ORFV- Humans** | 81.43% | 81.43% | 81.64%- 81.84% | 81.89%-82.09% | 81.14%-81.34% | 81.38%-81.59% | 81.57%-81.78% | 81.69%-81.89% |
| MN316550.1; MN316552.1; MN316553.1; MN316555.1  **ORFV - Muskoxen** | 80.18%-80.91% | 80.18%-80.91% | 80.65%-80.90% | 80.65%-81.33 | 80.18%-80.68% | 80.18%-80.88% | 80.18%-80.75% | 80.18%-80.88% |
| MH169570.1; MH169571.1  **BPSV - Humans** | 81.63% | 81.63% | 81.64% | 81.69% | 81.43% | 81.80% | 81.95% | 82.06% |

**Supplementary Table 2.** The BLAST nucleotide identity percentage between the sequences of the *DNA polymerase* gene obtained in this study (*) and corresponding parapoxvirus sequences from pinniped and terrestrial hosts published in GenBank database. Highlighted are the nucleotide identity percentages between the Steller sea lion AY952942.1 and the sequences obtained in this study.
